# Optimization of rooster semen preservation: a comparative study of extender types and antioxidant supplementation strategies during cold storage^☆^

**DOI:** 10.1016/j.psj.2025.105137

**Published:** 2025-04-04

**Authors:** Ruthaiporn Ratchamak, Supakorn Authaida, Thirawat Koedkanmark, Himalai Saiyamanon, Wuttigrai Boonkum, Vibuntita Chankitisakul

**Affiliations:** a The Research and Development Network Center of Animal Breeding and Omics, Khon Kaen University, Khon Kaen 40002, Thailand; b Department of Animal Science, Faculty of Agricultural, Khon Kaen University, Khon Kaen 40002, Thailand

**Keywords:** Semen preservation, Extender, Antioxidant, Cooling preservation, Sperm quality

## Abstract

This study evaluated the effect of semen extenders and antioxidant supplementation strategies on the quality and fertility of Thai native rooster semen during cold storage. In Experiment 1, four extenders (EK, IGGKPh, NaCl, and NCAB) were evaluated for their ability to preserve sperm motility, viability, lipid peroxidation (MDA), and oxidative stress (reactive oxygen species; ROS) over 24 h at 5°C. EK and IGGKPh extenders yielded superior motility (68.72% and 70.27%) and viability (91.96% and 91.97%) while minimizing MDA (0.93 and 1.12 µmol) and ROS (14.44% and 14.04%) compared to NaCl (motility: 53.14%, viability: 82.09±3.15, MDA: 1.59 µmol; P < 0.01). Experiment 2 examined the effects of dietary selenium (0.6 ppm), extender-based glutathione (GSH) supplementation (2mM), and their combination across all extenders. Antioxidant treatments, particularly GSH and its combination with selenium, improved motility, viability, and oxidative stability (P < 0.05). At 24 h, GSH-enriched groups using EK and IGGKPh exhibited enhanced motility (67.90% and 71.49%) compared to the controls (57.97% and 55.09%). In the NaCl group, GSH and combined treatments significantly improved motility at 12 h (50.83% and 50.01%, respectively) compared to the control (40.80%; P < 0.01). Selenium also increased sperm concentration (4.24 ± 0.55 × 10⁹ vs. 2.46 ± 0.22 × 10⁹ spz/mL; P < 0.01). Experiment 3 fertility outcomes using EK extender and the same antioxidant treatments from Experiment 2. Fertility rates were significantly higher in the GSH (70.38%) and combined (69.16%) groups than in the control (54.26%; P < 0.01), while hatchability remained unaffected (P > 0.05).In summary, the composition of the extender and antioxidant strategy has a significant influence on semen quality and fertility. The EK and IGGKPh extenders, especially when supplemented with GSH, offered superior protection, and combining dietary selenium with GSH yielded synergistic benefits for semen preservation and reproductive success.

## Introduction

Artificial insemination (AI) is crucial for efficient poultry breeding, particularly in turkey production, where size differences between males and females hinder natural mating (Mohan et al., 2018). Although less commonly adopted in commercial chicken production due to labor intensity, AI is gaining relevance in Thai native chicken farming for enhancing reproductive efficiency and conserving valuable genetic resources (Pimprasert et al., 2023). However, its success relies on the ability to maintain semen quality during storage, which declines rapidly within 24 h post-collection due to oxidative stress (Chankitisakul et al., 2022). This imbalance between reactive oxygen species (ROS) and endogenous antioxidant defenses leads to lipid peroxidation, comprising membrane integrity, motility, and fertilization potential (Rehman et al., 2018; Silvestre et al., 2021).

Semen extenders are essential for maintaining sperm motility and viability during cold storage. Effective extenders mimic the pH and osmolality of seminal plasma while supplying energy, stabilizing membranes, and reducing oxidative damage (Iaffaldano et al., 2005). Among the most promising formulations are IGGKPh (Surai and Wishart, 1996; Lukaszewicz, 2000), EK (Łukaszewicz, 2000), and NCAB (Chankitisakul et al., 2022), which offer superior protection compared to simple saline (NaCl), widely used by farmers for its low cost and availability. Despite these developments, comparative data on how different extenders influence oxidative stress—particularly within the critical first 24 h of storage—remain limited. Measuring malondialdehyde (MDA), a key biomarker of lipid peroxidation, provides valuable insight into the oxidative stability conferred by each extender.

Antioxidants are widely recognized as effective in preserving semen quality by neutralizing ROS (Bansal and Bilaspuri, 2010; Partyka and Niżański, 2021). Two principal strategies are employed: dietary supplementation and direct addition to extenders. Dietary selenium benefits older roosters and those under heat stress by reducing oxidative damage in both fresh and cryopreserved samples (Authaida et al., 2023). Combined with vitamin E, selenium enhances sperm count, motility, and viability (Ebeid, 2012), while vitamins C and E have been shown to improve fertility under heat stress (Attia et al., 2018). Phytogenic supplements, such as ginseng, also reduce lipid peroxidation and enhance fertility (Ratchamak et al., 2024).

Alternatively, antioxidant supplementation directly into extenders provides immediate oxidative protection during semen handling and storage. For example, coenzyme Q10 and soy phosphatidylcholine improved longevity in rooster semen (Nath et al., 2015), while citrus juice-based extenders enhanced sperm motility (Jimoh et al., 2021). Recent studies using *Eurycoma longifolia*, aloe vera gel, and glutathione (GSH) have demonstrated improved sperm integrity, lower MDA levels, and increased fertilization potential during chilled storage (Koedkanmark et al., 2024; Pimpa et al., 2024; Masoudi et al., 2020).

While antioxidant strategies have been extensively studied, direct comparisons between dietary and extender-based supplementation, particularly in combination with various extenders, remain scarce. This study addresses this gap by evaluating how extender composition and antioxidant delivery strategies interact to influence semen preservation in Thai native roosters. Using a three-experiment approach, Experiment 1 compared four semen extenders (NaCl, IGGKPh, EK, and NCAB) for their effects on sperm motility, viability, lipid peroxidation (MDA), and oxidative stress (ROS) during 24-hour cold storage at 5°C. Experiment 2 evaluated whether dietary selenium and extender-based GSH supplementation, individually or in combination, could further improve semen quality when applied across these extenders. To assess the functional relevance of these antioxidant strategies, Experiment 3 evaluated fertility and hatchability rates following insemination with chilled semen diluted in the best-performing extender from Experiment 1 and treated with each of the antioxidant regimens. By integrating extender formulation with dual antioxidant strategies and validating their impact on reproductive outcomes, this study provides new insights into semen preservation and practical applications for poultry AI programs.

## Materials and methods

The Institutional Animal Care and Use Committee approved the experimental procedures based on the Ethics of Animal Experimentation Guidelines of the National Research Council of Thailand (Record no. IACUC-KKU-130/67; Reference no. 660201.2.11/875 [126)).

### Chemicals and reagents

Selenium was obtained from ECONOMASE Alltech, Inc. (Nicholasville, KY, USA). GSH was purchased from Sigma-Aldrich (St. Louis, MO, USA; product number: G4251). The Muse® Oxidative Stress Kit was obtained from Merck KGaA (Darmstadt, Germany; Cat. No. MCH100111). All other chemicals and reagents were purchased from Sigma-Aldrich unless otherwise noted.

### Animals and management

A total of 30 Thai native roosters (*Pradu Hang Dum*), aged 52 to 72 weeks, and 28 Thai native hens (*Kaen Tong*), aged approximately one year, were used in this study. All birds were housed individually in battery cages (60 × 45 × 45 cm³) under an open-house system with natural daylight exposure for approximately 12 h per day.

Roosters were fed 130 g/day and hens 110 g/day of commercial breeder feed (Betagro Company Limited, Bangkok, Thailand), which was appropriate for their respective production stages. Fresh water was provided *ad libitum* to all birds.

Both sexes were managed under identical housing, feeding schedules, and environmental conditions to ensure consistency across the experimental period.

### Extender preparation

Four semen extenders were prepared according to the compositions outlined in Table 1. All reagents were weighed using an analytical balance with a readability of 0.1 mg, and the solutions were prepared under sterile conditions to prevent contamination. Each component was dissolved in distilled water at room temperature (∼25°C) under continuous stirring until complete dissolution was achieved. The final volume was adjusted to 100 mL. For pH standardization, the extenders were monitored using a calibrated pH meter (Checker®, Hanna Instruments, Bangkok, Thailand). If necessary, pH adjustments were made using either 0.1 M NaOH or 0.1 M HCl to achieve the target values specified in Table 1. Osmolality was measured using an osmometer (Wescor Inc., Logan, UT, USA) to ensure consistency with physiological conditions. Each extender was filtered through a 0.22 µm membrane filter for sterilization and stored overnight at 4°C prior to use.

**Table 1:** Composition of the four semen extenders used in this study.

Constituents	NaCl (g)	IGGKPh (g)	EK (g)	NCAB (g)
NaCl	0.90			
Potassium citrate monohydrate		0.14	0.14	0.10
Sodium glutamate		1.40	1.40	1.50
Sodium hydrogen phosphate		0.98		0.50
Sodium dihydrogen phosphate		0.21		0.05
Magnesium acetate				0.07
Sodium acetate				0.12
Glucose		0.90	0.70	1.00
Inositol		0.90	0.70	
Fructose			0.20	1.00
Polyvinylpyrrolidone			0.10	
Protamine sulfate			0.02	
Anhydrous sodium hydrogen phosphate			0.98	
Anhydrous sodium dihydrogen Phosphate			0.21	
D-Serine				0.04
mOsm	308	460	400	410
pH	5.5	7.8	7.8	9.6
Distilled water (mL)	100	100	100	100

Formulations were adapted from the following sources: Bootwalla and Miles (1992) for NaCl; Surai and Wishart (1996) for IGGKPh; Łukaszewicz (2000) for EK; and Chankitisakul et al. (2022) for NCAB.

### Semen sample collection and processing

Semen was collected weekly from each rooster by dorso-abdominal massage. Each ejaculate was collected into a 1.5-mL microtube containing 0.1 mL of one of four extenders: NaCl, IGGKPh, EK, and NCAB to prevent dehydration and osmotic stress. Care was taken to avoid contamination of semen with cloacal products such as feces, urates, and transparent fluids.

After collection, semen samples were immediately placed in a foam-insulated container and transported to the laboratory within 20 min under controlled conditions of 22–25°C to prevent temperature fluctuations. The transport container was not exposed to direct sunlight to minimize thermal shock. Upon arrival, semen samples were gently homogenized by inversion three times to ensure uniform dilution before further processing.

### Assessment of fresh semen quality

Fresh semen quality was assessed by evaluating semen volume, sperm motility, viability, and concentration. The semen volume was measured using a tuberculin syringe (Nipro Company Limited, Phra Nakhon Si Ayutthaya, Thailand). The motility of a coverslip-free semen sample was subjectively scored on a scale of 0 to 5 (0 = immotile; 5 = > 90% progressively motile sperm exhibiting rapid wave formation) using light microscopy (400 × magnification). Samples with a score > 3.5 were pooled and further evaluated before being divided into each extender group. Sperm viability and concentration were determined using pooled semen samples. Viability was determined using eosin-nigrosin staining. At least 300 sperm were assessed per sample (1000 × magnification) to determine the proportion of live (unstained) and dead (eosin-stained) sperm. Sperm concentration (× 10⁹ sperm/mL) was determined using a hemocytometer after dilution (1:1000 in 4% NaCl) and light microscopy (400 × magnification).

All assessments were performed in duplicate by the same experienced operator under blinded conditions to minimize bias. In brief, all semen samples were assigned randomly generated alphanumeric codes immediately after dilution with their respective extenders. These codes concealed the identity of the treatment group from the evaluator. An independent laboratory technician, who was not involved in the analysis, managed the sample labeling and maintained the treatment key throughout the evaluation period. The observer who assessed motility, viability, and other parameters remained blinded to treatment allocation until after all data were recorded and statistically analyzed.

### Semen dilution and semen storage

After assessing the pooled semen quality, semen samples were extended using the different semen extenders (1:3, v/v) according to the experimental design. The final concentration of the extended semen was approximately 100-150 × 10^6^ sperm/dose. The diluted semen samples were cooled from 25°C to 5°C for 1 hour before being stored at 5°C for further processing, as per the experimental design.

### Experimental design

Experiment 1 ***Effect of Extender Type (NaCl, IGGKPh, EK, and NCAB) and Storage Duration on Thai Native Rooster Semen Quality, Lipid Peroxidation, and Oxidative Stress during Cold Storage at 5°C.***

This experiment investigated the effects of four different extenders, NaCl, IGGKPh, EK, and NCAB, on semen quality, lipid peroxidation, and oxidative stress during 24 h of cold storage at 5°C. Semen samples were pooled and evenly divided into four aliquots, each diluted with a different extender. Semen quality parameters, including motility, viability, MDA, and ROS, were assessed at five time points: 0, 6, 12, 18, and 24 h post-extension and storage (T0, T6, T12, T18, and T24). The experiment was conducted in three replicates.

Experiment 2 ***Comparison of Antioxidant Supplementation Strategies (Dietary Selenium vs. Extender GSH) on Semen Quality in Thai Native Roosters.***

This experiment evaluated the individual and combined effects of dietary selenium supplementation and GSH enrichment of extenders on semen quality, lipid peroxidation, and oxidative stress during storage at 5°C. Four treatment groups were established: (1) Control (no supplementation); (2) Dietary selenium: 0.6 ppm selenium added to the diet (Authaida et al., 2023); (3) Extender GSH: 2 mM GSH added to the semen extender (Masoudi et al., 2020); and (4) Combined Supplementation: dietary selenium (0.6 ppm) and extender GSH (2 mM). Semen samples were collected weekly, with four replicates per treatment group. Semen quality parameters (motility, viability, lipid peroxidation, and oxidative stress) were assessed at 0, 12, and 24 h of storage at 5°C (T0, T12, T24).

Experiment 3 ***Evaluation of Fertility Following Antioxidant Supplementation and Cold Storage of Rooster Semen***

Following the semen quality assessment in Experiment 2, this experiment aimed to evaluate the effect of dietary selenium, extender-based GSH, and their combination on fertility outcomes after 24-hour semen storage at 5°C. Based on the results of Experiment 1, the EK extender—identified as the most effective in preserving semen quality—was selected as the base diluent for all treatment groups.

Semen samples from each antioxidant treatment group (Control, Dietary Selenium, Extender GSH, and Combined Supplementation) were diluted with the selected extender and stored at 5°C for 24 h before insemination. Thai native hens were inseminated once weekly with semen from their respective treatment groups. Fertility and Hatchability were recorded.

### Assessment of stored semen

Semen quality was evaluated in terms of total motility and viability. Total motility was assessed using light microscopy at 400 × magnification following dilution of 5 µL of semen with 100 µL of the respective extender. Viability was evaluated using the same procedure as described for fresh semen samples.

### Lipid peroxidation

Lipid peroxidation was assessed by measuring MDA levels using a thiobarbituric acid reactive substances assay. Semen samples were adjusted to a concentration of 250 × 10⁶ spz/mL and incubated with 0.25 mL of 1 mM ascorbic acid and 0.25 mL of 0.2 mM ferrous sulfate at 37°C for 60 min. Subsequently, 1 mL of 15% (w/v) trichloroacetic acid and 1 mL of 0.375% w/v thiobarbituric acid were added. The mixture was boiled at 100°C for 10 min to facilitate the reaction, which was then terminated by cooling to 4°C. Samples were centrifuged at 4000 × g for 10 min at 4°C. The MDA concentration (nmol/mL) in the supernatant was determined by ultraviolet-visible spectrophotometry at 532 nm using a Specord 250 Plus spectrophotometer (Analytikjena, Jena, Germany), as described by Ratchamak et al. (2023).

### Assessment of oxidative stress (ROS)

Oxidative stress was assessed by quantifying intracellular superoxide radicals using the Muse® Oxidative Stress Kit. Semen samples were prepared at a final concentration of 1 × 10⁶ sperm/mL in 1 × assay buffer. The oxidative stress reagent was first diluted 1:100 in 1 × assay buffer to create an intermediate solution, then further diluted 1:80 to prepare the working solution. A 10 µL aliquot of the semen sample was incubated with 190 µL of the working solution at 37°C for 30 min. After thorough mixing, samples were analyzed using the Muse® Cell Analyzer (Luminex Corporation, Texas, USA) to differentiate between ROS-negative (viable) and ROS-positive cells.

### Fertility test

Fertility was evaluated by inseminating Thai native layer hens once per week with cooled semen from each treatment group. Each hen received 0.1 mL of semen containing 100–150 × 10⁶ sperm per dose. Seven hens were assigned to each treatment group. Inseminations were performed between 3:00 p.m. and 5:00 p.m., and a single trained individual carried out all procedures to minimize variability due to handling. The insemination schedule was repeated once weekly over four consecutive weeks.

Eggs were collected from Day 2 after the first insemination until Day 8 following the final insemination. During this period, eggs were stored on plastic trays at a controlled ambient temperature of 22–25°C prior to incubation. Fertility was determined by candling on Day 7 of incubation to detect embryonic development. The fertility rate was calculated as the percentage of fertile eggs relative to the total number of incubated eggs. The hatchability rate was defined as the percentage of hatched chicks out of the total number of fertile eggs.

### Statistical analysis

Data from all experiments were first tested for normality using the Shapiro–Wilk test, and homogeneity of variances was assessed using Levene's test. Outliers were identified and excluded accordingly. A general linear model (GLM) was applied to analyze the data using analysis of variance (ANOVA), followed by Tukey's post hoc test to determine pairwise differences between treatment means.

Experiment 1 employed a split-plot design within a completely randomized framework, with extender types as the main plot and storage time as the subplot. Experiments 2 and 3 used a completely randomized design to evaluate the effects of dietary selenium, GSH-enriched extenders, and their combination on semen quality (Experiment 2) and on fertility and hatchability outcomes (Experiment 3).

Results were considered statistically significant at *P* < 0.05 and are presented as mean ± standard error (SE). All statistical analyses were performed using SAS software version 9.1 (SAS Institute Inc., Cary, NC, USA).

## Results

Experiment 1 ***Effect of Extender Type (NaCl, IGGKPh, EK, and NCAB) and Storage Duration on Semen Quality, Lipid Peroxidation, and Oxidative Stress During Cold Storage at 5°C***

Table 2 summarizes the semen characteristics observed in fresh semen samples collected from Thai native roosters. ANOVA revealed significant effects of both storage duration and extender type (*P* < 0.01) on all semen quality parameters, including motility, viability, MDA, and ROS. However, the interaction between storage time and extender type was not statistically significant.

**Table 2:** Fresh semen characteristics of Thai native roosters.

Semen characteristic	Mean±SE
Ejaculate volume (mL)	0.33±0.03
Sperm concentration/mL (× 10^9^ spz/mL)	3.31±0.18
Sperm motility (0-5)	3.93±0.11
Sperm viability (%)	89.69±0.92
Total sperm per ejaculate (× 10^9^ spz)	1.12±0.15

SE, standard error

Table 3 shows the effects of storage duration on sperm quality. Both motility and viability decreased significantly over time (*P* < 0.0001). A sharp decline in motility was observed from the initial value at T0 (81.1 ± 2.45%) to 12 h post-storage (56.95 ± 3.65%; *P* < 0.01), continuing to fall to 49.43 ± 3.75% at 24 h. While viability remained statistically unchanged up to 18 h (*P* > 0.05), a significant reduction was noted at 24 h (79.26 ± 3.84%; *P* < 0.01).

**Table 3:** Effect of storage time on sperm quality of Thai native roosters (Pradu Hang Dam).

Parameter	n	T0	T6	T12	T18	T24	*P*-value
Motility (%)	12	81.12±2.45^a^	77.28±1.29^a^	56.95±3.65^b^	54.10±4.09^b^	49.43±3.75^b^	<0.0001
Viability (%)	12	90.30±0.73^a^	94.00±0.73^a^	89.98±2.33^a^	87.54±1.89^ab^	79.26±3.84^b^	0.0010
MDA (µmol)	12	0.97±0.07^c^	1.01±0.09^bc^	1.16±0.15^bc^	1.28±0.15^ab^	1.52±0.13^a^	<0.0001
ROS (%)	12	9.32±2.28^c^	13.08±2.12^bc^	17.05±2.24^b^	23.80±2.34^a^	28.24±1.52^a^	<0.0001

MDA, malondialdehyde; ROS, reactive oxygen species. Values are presented as mean ± standard error (SE). Values within rows with different superscript letters (a–c) differ significantly (P < 0.05).

Conversely, both MDA and ROS levels increased significantly with storage time (*P* < 0.01). MDA levels began to increase at 18 h (1.28 ± 0.15 µmol), peaking at 24 h (1.52 ± 0.13 µmol), compared to the baseline value at T0 (0.97 ± 0.07 µmol). ROS levels showed a significant increase as early as 12 h (17.05 ± 2.24%; *P* < 0.01), reaching 28.24 ± 1.52% at 24 h, up from 9.32 ± 2.28% at T0.

Table 4 shows the effects of different extenders on semen quality. Significant differences were observed among extenders for all measured parameters (*P* < 0.01). The highest motility was recorded in the IGGKPh (70.27 ± 3.73%) and EK (68.72 ± 4.00%) groups, which were not statistically different from each other (*P* > 0.05), but both were significantly higher than that of the NaCl group (53.14 ± 4.04%). The NCAB group displayed intermediate motility (62.98 ± 4.54%).

**Table 4:** Effect of semen extenders on sperm quality of Thai native roosters (Pradu Hang Dam).

Parameter	n	EK	IGGKPh	NaCl	NCAB	P-value
Motility (%)	15	68.72±4.00^ab^	70.27±3.73^a^	53.14±4.04^c^	62.98±4.54^b^	<0.0001
Viability (%)	15	91.96±0.68^a^	91.97±0.86^a^	82.09±3.15^b^	86.82±2.46^ab^	0.0036
MDA (µmol)	15	0.93±0.11^b^	1.12±0.07^b^	1.59±0.13^a^	1.13±0.08^b^	<0.0001
ROS (%)	15	14.44±2.37^c^	14.04±2.07^c^	24.81±2.29^a^	19.93±2.67^b^	0.0006

MDA, malondialdehyde; ROS, reactive oxygen species. Values are presented as mean ± standard error (SE). Values within rows with different superscript letters (a–c) differ significantly (P < 0.05).

Similarly, viability was highest in the IGGKPh (91.97 ± 0.86%) and EK (91.96 ± 0.68%) groups, both of which significantly outperformed the NaCl group (82.09 ± 3.15%; **P** < 0.01). MDA levels were lowest in the EK (0.93 ± 0.11 µmol) and IGGKPh (1.12 ± 0.07 µmol) groups, while the NaCl group showed the highest level (1.59 ± 0.13 µmol; *P* < 0.01). ROS levels followed a similar trend: the IGGKPh (14.04 ± 2.07%) and EK (14.44 ± 2.37%) groups showed significantly lower values, whereas the NaCl group had the highest ROS levels (24.81 ± 2.29%; *P* < 0.01). Although not the most effective, the NCAB extender consistently outperformed NaCl across all parameters.

**Fig. 1:**
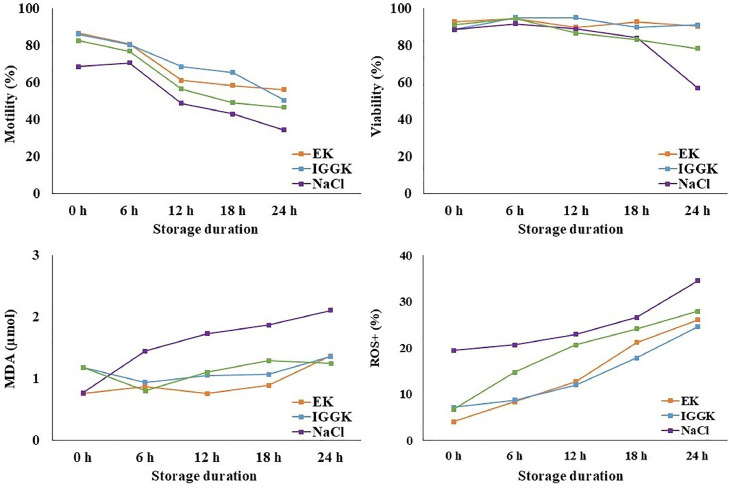
Effects of semen extender type and storage duration on rooster semen quality during cold storage at 5°C. Semen samples from Thai native roosters were diluted in three extenders: EK, IGGKPh, and NaCl, and evaluated at 0, 6, 12, 18, and 24 h of storage at 5°C.

Fig. 1 illustrates the effects of extender type and storage duration (0, 6, 12, 18, and 24 h at 5°C) on semen motility, viability, MDA, and ROS levels.

**Motility** declined in all groups over time, with notable differences in the extent of preservation. The NaCl group showed the lowest motility, dropping below 50% as early as 12 h (48.82%) and reaching 34.37% by 24 h. In contrast, the EK and IGGKPh groups maintained the highest motility at 24 h (56.21% and 50.55%, respectively), while the NCAB group performed moderate motility (46.61%).

**Viability** in the EK, IGGKPh, and NCAB groups remained consistently high, exceeding 85% throughout the first 18 h of storage. In contrast, the NaCl group began to show a noticeable decline in viability after 18 h, dropping from 84.06% to just 57.11% at 24 h. By this final time point, the NaCl group exhibited the lowest viability, significantly lower than that observed in the IGGKPh (91.17%) and EK (90.40%) groups. The NCAB group showed intermediate viability at 24 h (78.35%), performing better than NaCl but not as well as EK and IGGKPh.

**MDA and ROS levels** increased in all groups over time, indicating a progressive increase in oxidative stress. The NaCl group consistently exhibited the highest oxidative markers, with MDA increasing from 0.77 µmol at T0 to 2.11 µmol at 24 h, and ROS increasing from 19.45% to 34.48% over the same period. EK and NCAB exhibited the lowest oxidative increases, with MDA values at 1.37 µmol and 1.25 µmol, and ROS at 26.01% and 27.90%, respectively, at 24 h. IGGKPh provided moderate oxidative protection, with final MDA and ROS levels of 1.36 µmol and 24.59%, respectively.

Although all extenders showed a similar upward trend in oxidative stress over time, the NaCl group exhibited the steepest increases, particularly in ROS levels. This underscores its limited antioxidant capacity during storage. EK and NCAB provided the most effective protection against oxidative damage, followed closely by IGGKPh.

Experiment 2 ***Comparison of Antioxidant Supplementation Strategies (Dietary Selenium vs. Extender GSH) on Semen Quality in Thai Native Roosters***

The effects of dietary selenium supplementation on fresh semen characteristics are presented in Table 5. Sperm concentration was significantly higher in the selenium-treated group (4.24 ± 0.55 × 10⁹ spz/mL) compared to the control (2.46 ± 0.22 × 10⁹ spz/mL; *P* = 0.0407). Similarly, the total sperm count per ejaculate was significantly greater in the treatment group (1.42 ± 0.01 × 10⁹) than in the control group (0.79 ± 0.01 × 10⁹; *P* = 0.0083). No significant differences were observed in ejaculate volume, sperm motility score, or viability, although slightly higher mean values were recorded in the treatment group.

**Table 5:** Fresh semen characteristics of Thai native roosters with (Treatment) or without (Control) dietary selenium supplementation.

**Semen characteristic**	**Control**	**Treatment**	**P-value**
Ejaculated volume (mL)	0.32±0.02	0.34±0.01	0.6349
Sperm concentration/mL (× 10^9^ spz/mL)	2.46±0.22^a^	4.24±0.55^b^	0.0407
Sperm motility (0-5)	3.77±0.23	4.02±0.06	0.3701
Sperm viability (%)	93.30±0.71	95.91±2.00	0.4011
Total sperm per ejaculate (× 10⁹ spz)	0.79±0.01^a^	1.42±0.01^b^	0.0083

Values are presented as mean ± standard error (SE). Values within rows with different superscript letters (a, b) indicate statistically significant differences (P < 0.05).

The effects of dietary selenium, GSH-enriched extenders, and their combination during cold storage are detailed in Table 6, Table 7, Table 8, Table 9, which compare sperm quality across different extender types (EK, IGGKPh, NaCl, and NCAB).

**Table 6:** Effect of antioxidant supplementation strategies on semen quality parameters using the EK extender following 24 h of storage at 5°C.

Parameter	Control	Dietary(+Se)	Extender (+GSH)	Combination	SEM	P-value
Motility (%)						
T0	77.85	76.46	78.20	74.59	1.25	0.8252
T12	69.53	64.42	72.35	68.43	1.70	0.4835
T24	57.97^b^	62.76^ab^	67.90^a^	63.62^a^	2.35	0.0083
Viability (%)						
T0	92.33	92.45	89.04	92.80	0.99	0.1119
T12	82.92	88.94	83.93	81.48	1.57	0.4357
T24	69.05^b^	78.24^a^	81.90^a^	78.19^a^	1.72	0.0081
MDA (µmol)						
T0	1.14	1.13	0.82	1.00	0.08	0.3630
T12	1.27	0.91	1.09	1.06	0.06	0.1790
T24	1.70^a^	1.36^b^	1.35^b^	1.10^b^	0.08	0.0032
ROS (%)						
T0	18.35	18.61	22.70	19.75	1.95	0.7478
T12	26.23	31.77	23.84	28.58	3.49	0.4443
T24	46.38^a^	34.58^ab^	25.25^a^	27.90^a^	3.53	0.0177

Se = selenium; GSH = glutathione; MDA = malondialdehyde; ROS = reactive oxygen species; SEM = standard error of the mean. Timepoints: T0 = 0 h, T12 = 12 h, T24 = 24 h of semen storage at 5°C.Values within the same row with different superscript letters (a, b) differ significantly (P < 0.05).

**Table 7:** Effect of antioxidant supplementation strategies on semen quality parameters using the IGGKPh extender following 24 h of storage at 5°C.

Parameter	Control	Dietary(+Se)	Extender (+GSH)	Combination	SEM	P-value
Motility (%)						
T0	86.50	79.42	80.44	82.97	1.31	0.1472
T12	69.36	70.53	68.83	66.64	1.51	0.7901
T24	55.09^b^	64.00^ab^	71.49^a^	70.48^a^	2.31	0.0114
Viability (%)						
T0	93.87	94.31	92.60	93.84	0.50	0.1019
T12	86.91	88.43	86.29	83.59	0.97	0.2281
T24	74.12^b^	83.90^a^	82.38^a^	81.06^a^	1.39	0.0007
MDA (µmol)						
T0	1.08	0.89	0.92	1.14	0.05	0.4714
T12	1.27	1.12	1.32	1.27	0.04	0.1648
T24	1.31^a^	1.09^b^	1.14^b^	1.09^b^	0.04	0.0027
ROS (%)						
T0	16.81	14.42	17.08	19.75	1.61	0.4112
T12	23.51	17.24	19.51	22.46	3.19	0.8654
T24	39.48^a^	29.87^b^	27.94^b^	27.74^b^	2.05	0.0091

Se = selenium; GSH = glutathione; MDA = malondialdehyde; ROS = reactive oxygen species; SEM = standard error of the mean. Timepoints: T0 = 0 h, T12 = 12 h, T24 = 24 h of semen storage at 5°C.Values within the same row with different superscript letters (a, b) differ significantly (P < 0.05).

**Table 8:** Effect of antioxidant supplementation strategies on semen quality parameters using the NaCl extender following 24 h of storage at 5°C.

Parameter	Control	Dietary(+Se)	Extender (+GSH)	Combinatio	SEM	P-value
Motility (%)						
T0	60.66	57.43	62.79	62.81	2.85	0.6806
T12	40.80^b^	45.95^ab^	50.83^a^	50.01^a^	1.38	0.0117
T24	30.97^b^	40.43^a^	42.13^a^	39.58^a^	1.56	0.0020
Viability (%)						
T0	93.46	92.14	92.52	90.09	0.61	0.0666
T12	87.55	85.24	85.97	80.04	1.60	0.4034
T24	73.41^b^	84.10^a^	83.12^a^	81.72^a^	1.22	0.0009
MDA (µmol)						
T0	1.37	1.30	1.30	1.17	0.05	0.3951
T12	1.73^a^	1.41^ab^	1.32^b^	1.25^b^	0.07	0.0205
T24	2.05^a^	1.58^b^	1.63^b^	1.51^b^	0.06	0.0038
ROS (%)						
T0	20.49	19.26	20.17	19.75	1.01	0.9113
T12	27.59	23.88	23.46	28.05	2.77	0.8547
T24	45.45^a^	38.88^ab^	29.43^c^	34.80^bc^	3.03	0.0030

Se = selenium; GSH = glutathione; MDA = malondialdehyde; ROS = reactive oxygen species; SEM = standard error of the mean. Timepoints: T0 = 0 h, T12 = 12 h, T24 = 24 h of semen storage at 5°C.Values within the same row with different superscript letters (a, b) differ significantly (P < 0.05).

**Table 9:** Effect of antioxidant supplementation strategies on semen quality parameters using the NCAB extender following 24 h of storage at 5°C.

Parameter	Control	Dietary(+Se)	Extender (+GSH)	Combination	SEM	P-value
Motility (%)						
T0	77.07	76.74	75.01	74.12	1.38	0.7805
T12	64.41	62.98	62.99	63.90	1.37	0.9581
T24	61.41	63.20	60.58	62.61	1.98	0.9598
Viability (%)						
T0	91.36	90.63	92.83	93.58	1.01	0.6071
T12	81.57	83.44	87.90	85.19	1.47	0.3836
T24	70.07^b^	80.33^a^	82.87^a^	81.35^a^	2.00	0.0059
MDA (µmol)						
T0	1.29^a^	1.19^ab^	1.18^ab^	1.14^b^	0.02	0.0269
T12	1.48^a^	1.32^ab^	1.18^b^	1.25^b^	0.06	0.0037
T24	1.64^a^	1.45^ab^	1.37^b^	1.32^b^	0.04	0.0089
ROS (%)						
T0	20.87	18.77	17.86	19.75	1.49	0.8480
T12	36.72^a^	24.58^b^	23.87^b^	24.02^b^	2.58	0.0225
T24	39.56^a^	38.35^a^	24.72^b^	24.51^b^	2.08	0.0002

Se = selenium; GSH = glutathione; MDA = malondialdehyde; ROS = reactive oxygen species; SEM = standard error of the mean. Timepoints: T0 = 0 h, T12 = 12 h, T24 = 24 h of semen storage at 5°C.Values within the same row with different superscript letters (a, b) differ significantly (P < 0.05).

### EK extender

At 24 h, sperm motility was significantly higher in the GSH-enriched and combination groups (67.90% and 63.62%, respectively) than in the control (57.97%; *P* = 0.0083). Viability was also significantly improved in the dietary selenium, GSH-enriched, and combination groups (78.24%, 81.90%, and 78.19%, respectively) compared to that in the control (69.05%; *P* = 0.0081). MDA and ROS levels were significantly lower in all antioxidant-treated groups, with the combination treatment yielding the lowest values for both MDA (1.10 µmol; *P* = 0.0032) and ROS (27.90%; *P* = 0.0177).

### IGGKPh extender

Sperm motility at 24 h was significantly improved in the GSH-enriched (71.49%) and combination (70.48%) groups compared to the control (55.09%; *P* = 0.0114). Similarly, viability was significantly higher in the dietary selenium, GSH-enriched, and combination groups (83.90%, 82.38%, and 81.06%, respectively) than in the control (74.12%; *P* = 0.0007). MDA levels were lower in all antioxidant-treated groups, with the combination group showing a reduction to 1.09 µmol versus 1.31 µmol in the control (*P* = 0.0027). ROS levels followed a similar trend, with significantly lower values in all antioxidant-treated groups; the combination group recorded the lowest ROS level at 27.74%, compared to 39.48% in the control (*P* = 0.0091).

### NaCl extender

Significant improvements in both motility and oxidative stress indicators were observed in the antioxidant-treated groups. At 12 h, motility was significantly higher in the GSH-enriched (50.83%) and combination (50.01%) groups than in the control (40.80%; *P* = 0.0117). MDA levels at this time point were also significantly reduced in the antioxidant-treated groups, with values of 1.25 µmol (combination) and 1.32 µmol (GSH-enriched) versus 1.73 µmol in the control (*P* = 0.0205).

At 24 h, motility remained significantly higher in all antioxidant-treated groups: 40.43% (selenium), 42.13% (GSH-enriched), and 39.58% (combination), compared to 30.97% in the control (**P** = 0.0020). Viability at 24 h showed a similar trend, with antioxidant-treated groups demonstrating significantly higher values (84.10%, 83.12%, and 81.72%) relative to the control (73.41%; *P* = 0.0009). MDA levels were lowest in the combination group (1.51 µmol), significantly reduced compared to the control (2.05 µmol; *P* = 0.0038). ROS levels were also significantly lower, with the GSH-enriched group showing the lowest value (29.43%) compared to 45.45% in the control (*P* = 0.0030).

### NCAB extender

Although no significant differences in motility were observed across treatments at 24 h, viability was significantly enhanced in all antioxidant-treated groups. The dietary selenium (80.33%), GSH-enriched (82.87%), and combination (81.35%) groups showed higher viability than the control (70.07%; *P* = 0.0059). MDA levels were also significantly reduced in the combination group (1.32 µmol) compared to the control (1.64 µmol; *P* = 0.0089). ROS levels followed a similar pattern, with significant reductions in the GSH-enriched (24.72%) and combination (24.51%) groups relative to the control (39.56%; *P* = 0.0002).

Overall, across all extender types, antioxidant supplementation significantly improved sperm motility, viability, and oxidative stress parameters, including MDA and ROS. The GSH-enriched and combination treatments consistently yielded the best results, particularly in terms of oxidative protection. The NaCl extender exhibited the greatest relative improvement with antioxidant intervention, highlighting its high susceptibility to oxidative stress. In contrast, the NCAB extender showed minimal variation in motility across treatments, though improvements in viability and oxidative markers were still observed.

Experiment 3 ***Evaluation of Fertility Following Antioxidant Supplementation and Cold Storage of Rooster Semen***

**Fig. 2:**
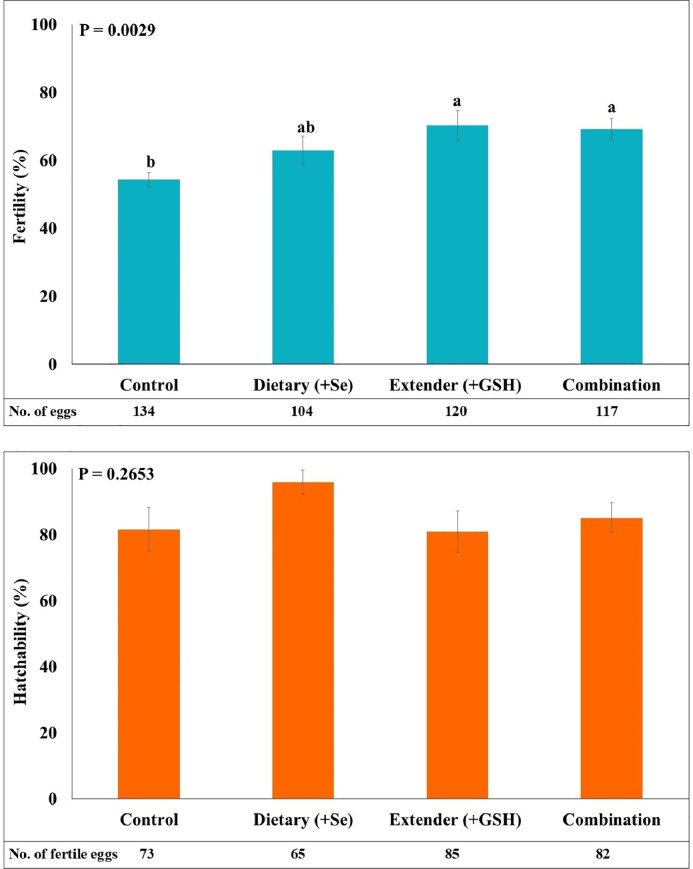
Effects of antioxidant supplementation strategies on fertility and hatchability percentages in Thai native roosters. Semen was stored for 24 h at 5°C using the EK extender and treated with four antioxidant strategies: Control (no supplementation), Dietary (+ Selenium), Extender (+ GSH), and Combination (Selenium + GSH). The total number of eggs or fertile eggs used in each group is indicated below each bar. Bars with different superscript letters (a, b) indicate significant differences (P < 0.05). Error bars represent the standard error of the mean (SEM).

The effects of dietary selenium supplementation, extender-based GSH enrichment, and their combination on fertility and hatchability rates are presented in Fig. 2. Antioxidant supplementation significantly improved the fertility rate (*P* < 0.01). Roosters whose semen was preserved with the GSH-enriched extender exhibited the highest fertility rate (70.38 ± 4.29%), followed closely by those receiving the combined dietary selenium and extender GSH treatment (69.16 ± 3.16%). Both treatments yielded significantly higher fertility rates than the control group (54.26 ± 2.02%; *P* < 0.01). The group receiving dietary selenium alone exhibited an intermediate fertility rate (62.87 ± 4.27%), which was not statistically different from either the control or the GSH-enriched groups.

Regarding hatchability, no statistically significant differences were observed among the treatment groups (*P* > 0.05).

## Discussion

This study provides a comprehensive evaluation of how extender composition and antioxidant supplementation strategies influence semen quality during cold storage in Thai native roosters. By comparing four semen extenders and incorporating two antioxidant approaches—dietary selenium and GSH enrichment of extenders—we demonstrate that these factors have both independent and synergistic effects on sperm preservation. Notably, the EK and IGGKPh extenders showed superior preservation of motility and viability, along with significantly lower oxidative stress markers (MDA and ROS levels), compared to NaCl, which consistently underperformed. Antioxidant supplementation, particularly GSH enrichment and its combination with dietary selenium, further enhanced sperm quality across all extenders, with the most pronounced benefits observed after 24 h of storage. Interestingly, the NaCl extender showed early declines in quality by 12 h, highlighting its heightened susceptibility to oxidative stress and stronger dependence on antioxidant intervention. However, its overall performance remained lower than that of the other extenders. These findings underscore the critical importance of optimizing both extender formulation and antioxidant delivery to enhance semen quality, particularly in extended storage scenarios, and provide valuable insights for practical application in poultry reproduction systems.

Experiment 1 revealed that the EK and IGGKPh extenders significantly outperformed NCAB and NaCl in preserving rooster sperm motility and viability during 24-hour cold storage. These results were supported by consistently lower levels of MDA and ROS, indicating superior protection against oxidative stress. The enhanced performance of EK and IGGKPh can be attributed to their optimized formulations, which include energy substrates (e.g., glucose), cryoprotective compounds, and buffering agents that collectively stabilize sperm membranes and reduce lipid peroxidation (Surai and Wishart, 1996; Łukaszewicz, 2000). A key component in both extenders is inositol, a naturally occurring antioxidant found in seminal plasma, which plays a critical role in osmotic regulation and membrane protection (Mann, 1953; Doğu et al., 2021). Additionally, EK contains polyvinylpyrrolidone, a non-antioxidant polymer that contributes to membrane stability and viscosity, indirectly enhancing sperm resilience to oxidative damage by reducing membrane fluidity and supporting other antioxidant actions (Partyka and Niżański, 2021).

NCAB, although not as effective as EK or IGGKPh, demonstrated intermediate preservation capacity, likely due to the inclusion of serine-based compounds with known antioxidant and cryoprotective properties (Chankitisakul et al., 2022). This may explain its ability to reduce ROS levels relative to NaCl, albeit less effectively than extenders with more comprehensive buffering and antioxidant systems.

In contrast, the simple saline solution (NaCl) consistently exhibited the poorest performance in preserving semen quality. Its lack of energy substrates, buffering agents, and antioxidant molecules led to a rapid decline in motility and viability, accompanied by significantly elevated oxidative damage indicators (MDA and ROS), which were detectable as early as 12 h post-storage. This rapid deterioration is consistent with previous reports on the susceptibility of sperm to deterioration in minimal media lacking protective compounds (Tvrdá et al., 2013; Chankitisakul et al., 2022). Notably, motility dropped below 50% by 12 h—a critical threshold often associated with reduced fertilization potential due to impaired sperm transport and survival within the female reproductive tract (Donoghue & Wishart, 2000). These findings underscore the inadequacy of NaCl for extended semen preservation and highlight the importance of using well-formulated extenders to maintain functional sperm quality during cold storage.

In Experiment 2, we investigated the effects of antioxidant supplementation, both dietary selenium and GSH enrichment in extenders, on semen quality during cold storage. Both strategies significantly improved preservation outcomes, with the most pronounced effects observed at 24 h post-storage. Enhancements were particularly notable in motility and viability, emphasizing the importance of oxidative protection during prolonged storage. Notably, when semen was stored in EK, IGGKPh, or NCAB, no significant differences were observed between control and antioxidant-treated groups at 0 or 12 h. This suggests that these extenders offer intrinsic antioxidant protection, likely due to the presence of inositol in EK and IGGKPh, and serine in NCAB, which may be sufficient to prevent early-stage oxidative damage.

In contrast, semen stored in the NaCl extender, which lacks essential buffering agents, energy substrates, and antioxidant components, showed a clear dependence on antioxidant supplementation. GSH and selenium treatments resulted in marked improvements, with motility increasing to over 50% in treated samples by 12 h, compared to only 40% in the control. These findings align with and extend previous work by Yaman et al. (2022), who observed a 42% reduction in sperm quality under similar storage conditions, indicating that our antioxidant interventions more effectively mitigated oxidative damage. Although NaCl is not ideal for long-term preservation, our findings suggest that it may serve as a cost-effective alternative for short-term use when combined with appropriate antioxidant strategies—an important consideration for poultry systems with limited access to advanced extenders.

Glutathione (GSH), a tripeptide composed of glutamate, cysteine, and glycine, plays a central role in antioxidant defense by neutralizing ROS and regenerating other antioxidants, such as vitamins C and E (Sonkusare and Chaturvedi, 2020). In this study, GSH-enriched extenders consistently demonstrated superior antioxidant efficacy, as evidenced by significantly lower MDA and ROS levels across all storage durations. This indicates improved membrane stability and reduced lipid peroxidation during cold storage. These results align with previous findings in avian species, which demonstrate that GSH supplementation aids in maintaining sperm motility, membrane integrity, and fertilization capacity during storage (Iswati et al., 2017; Masoudi et al., 2020; Zuha et al., 2023). Furthermore, antioxidant interventions in heat-stressed mammalian models, such as rabbits treated with vitamin E nano-emulsions, have similarly been shown to preserve testicular and sperm structural integrity (El-Raghi et al., 2024), supporting the broader relevance of antioxidant strategies in protecting male fertility under stress.

Selenium, an essential trace element, contributes to redox regulation as a cofactor for glutathione peroxidase, catalyzing the breakdown of hydrogen peroxide and lipid hydroperoxides (Marković et al., 2018; Wróblewski et al., 2024). In our study, dietary selenium supplementation improved motility, viability, and oxidative stress markers, findings consistent with those of prior studies (Ebeid, 2012; Authaida et al., 2023). Beyond its antioxidant role, selenium significantly enhanced sperm concentration, indicating support for spermatogenesis. It promotes seminiferous tubule development and Sertoli cell viability by regulating key reproductive genes, such as those encoding selenoprotein W and LH/choriogonadotropin receptors (Surai & Fisinin, 2014; Khalid et al., 2016; Chauychu-noo et al., 2021). These dual benefits are well-supported in livestock, where selenium—particularly in nanoparticle form—has been linked to enhanced testicular function, improved endocrine health, and increased sperm output (Abdelnour et al., 2021).

While both antioxidant strategies were beneficial, extenders enriched with GSH, either alone or in combination with dietary selenium, demonstrated superior efficacy compared to dietary selenium alone. This advantage may result from the continuous generation of ROS during cold storage, which is more efficiently mitigated through direct antioxidant supplementation in the extender medium. In contrast, dietary antioxidants may become metabolically unavailable or depleted over time, particularly beyond 24–36 h, as shown in studies involving *Kaempferia parviflora,* where antioxidant capacity diminished with prolonged storage (Authaida et al., 2024). Moreover, environmental challenges such as heat stress can suppress the activities of antioxidant enzymes, further exacerbating oxidative damage in semen (Sahin et al., 2023). These observations highlight the practical value of extender-based antioxidant delivery, which provides both immediate and sustained oxidative protection during storage.

Importantly, the combined use of dietary selenium and GSH-enriched extenders produced additive effects, yielding better semen quality compared to either strategy alone. This likely reflects their complementary mechanisms: GSH acts locally within the extender to directly scavenge ROS, while dietary selenium enhances endogenous antioxidant defenses by stimulating GSH peroxidase activity. Together, they offer a dual defense against both exogenous and endogenous oxidative stress, thereby maximizing sperm preservation during cold storage.

In Experiment 3, we confirmed the functional relevance of our semen quality findings by evaluating fertility outcomes following insemination with semen stored for 24 h in the EK extender under different antioxidant treatments. Fertility rates were significantly higher in the GSH-enriched (70.38%) and combination (69.16%) treatment groups compared to the control (54.26%; *P* < 0.01), confirming that antioxidant-mediated improvements in semen quality translate into enhanced reproductive performance. These improvements in fertility closely paralleled prior enhancements in motility, viability, and oxidative stability, suggesting that antioxidant-enriched semen retained greater fertilization capacity. Glutathione preserves membrane integrity by stabilizing thiol groups and scavenging ROS, thereby mitigating lipid peroxidation and maintaining the fluidity necessary for successful transit through the female reproductive tract (Donoghue & Donoghue, 1997; Zanloo et al., 2022). In parallel, dietary selenium supports mitochondrial redox homeostasis by enhancing glutathione peroxidase activity, contributing to the structural stability of sperm cells and their ability to maintain functionality during storage (Ebeid, 2009; Uzochukwu et al., 2025). These antioxidant effects are particularly relevant in avian species, where sperm must remain viable in the sperm storage tubules (SSTs) for prolonged periods. Consistent with previous studies (Masoudi et al., 2020; Wróblewski et al., 2024), improved oxidative resilience contributed to better fertilization outcomes under cold storage.

Interestingly, dietary selenium alone (62.87%) did not differ statistically from the control group, likely due to its reduced efficacy during prolonged storage, as observed in prior studies where selenium effects were limited after 24 h, which overwhelms the endogenous antioxidant capacity, particularly in the absence of direct antioxidant agents within the semen medium.(e.g., Meamar et al., 2016)

Notably, despite these improvements in fertility, hatchability rates remained statistically unchanged across treatments (*P* > 0.05), indicating that antioxidant supplementation primarily benefits pre-fertilization sperm function rather than post-fertilization embryo development. This further supports the idea that the observed fertility gains are directly attributable to improved sperm quality rather than secondary factors.

## Conclusions

The choice of semen extender plays a critical role in determining the success of cold storage, with formulations such as EK and IGGKPh demonstrating superior preservation of sperm motility, viability, and oxidative stability compared to simpler solutions like NaCl. Antioxidant supplementation within extenders, particularly with glutathione, proved more effective than dietary supplementation alone in mitigating oxidative stress and maintaining sperm quality during storage. Nonetheless, dietary selenium contributed significantly to improving sperm concentration, likely through its role in supporting spermatogenesis, thereby enhancing overall semen output.

Notably, the combination of dietary selenium and extender-based GSH supplementation produced additive effects, offering a synergistic approach that supports both sperm quality and quantity. This dual-benefits strategy is especially valuable for optimizing reproductive performance in rooster breeders, where both functional integrity and sperm output are essential. The benefits of this strategy were also evident at the functional level: fertility rates were significantly higher in the GSH-enriched and combined treatment groups, confirming that improvements in semen quality translate into greater reproductive success following artificial insemination.

Although NaCl was the least effective extender overall, our findings suggest that its performance can be substantially improved through antioxidant supplementation, making it a viable option for short-term semen storage in resource-limited settings. Future research should investigate the reformulation of low-cost extenders, such as NaCl, by incorporating buffering agents, energy substrates, and synergistic antioxidants to enhance their efficacy in preserving sperm function during cold storage.

## Declaration of competing interest

The authors declare the following financial interests/personal relationships, which may be considered as potential competing interests:

Ruthaiporn Ratchamak reports financial support was provided by Khon Kaen University (under the Postdoctoral Training for Frontier Research program from Khon Kaen University, Thailand [Grant No. PD2567-02]). If there are other authors, they declare that they have no known competing financial interests or personal relationships that could have appeared to influence the work reported in this paper.

## Appendix Supplementary materials

Image, application 1

## References

[bib0001] Abdelnour S.A., Alagawany M., Hashem N.M., Farag M.R., Alghamdi E.S., Hassan F.U., Bilal R.M., Elnesr S.S., Dawood M.A.O., Nagadi S.A., Elwan H.A.M., ALmasoudi A.G., Attia Y.A. (2021). Nanominerals: fabrication methods, benefits and hazards, and their applications in ruminants with special reference to selenium and zinc nanoparticles. Animals.

[bib0003] Attia Y.A., Abd El-Hamid A.E., Abdallah A.A., Berikaa M.A., El-Gandy M.F., Sahin K., Abou-Shehema B.M. (2018). Effect of betaine, vitamin C and vitamin E on egg quality, hatchability, and markers of liver and renal functions in dual-purpose breeding hens exposed to chronic heat stress. Europ. Poult. Sci.

[bib0005] Authaida S., Ratchamak R., Boonkum W., Chankitisakul V. (2023). Increasing sperm production and improving cryosurvival of semen in aged Thai native roosters as affected by selenium supplementation. Anim. Biosci..

[bib0006] Authaida S., Chankitisakul V., Ratchamak R., Pimpa J., Koedkanmark T., Boonkum W., Khonmee J., Tuntiyasawasdikul S. (2024). The effect of Thai ginger (Kaempferia parviflora) extract orally administration on sperm production, semen preservation, and fertility in Thai native chickens under heat stress. Poult. Sci..

[bib0007] Bansal A.K., Bilaspuri G.S. (2010). Impacts of oxidative stress and antioxidants on semen functions. Vet. Med. Int..

[bib0008] Bootwalla S.M., Miles R.D. (1992). Development of diluents for domestic fowl semen. Worlds Poult. Sci. J..

[bib0009] Chankitisakul V., Boonkum W., Kaewkanha T., Pimprasert M., Ratchamak R., Authaida S., Thananurak P. (2022). Fertilizing ability and survivability of rooster sperm diluted with a novel semen extender supplemented with serine for practical use on smallholder farms. Poult. Sci..

[bib0010] Chauychu-Noo N., Thananurak P., Boonkum W., Vongpralub T., Chankitisakul V. (2021). Effect of organic selenium dietary supplementation on quality and fertility of cryopreserved chicken sperm. Cryobiology.

[bib0011] Doğu Z., Şahinöz E., Aral F., Koyuncu İ., Yüksekdağ Ö. (2021). Effects of inositol supplementation in sperm extender on the quality of cryopreserved mesopotamian catfish (*Silurus triostegus*, H. 1843) sperm. Animals.

[bib0012] Donoghue A.M., Donoghue D.J. (1997). Effects of water- and lipid-soluble antioxidants on turkey sperm viability, membrane integrity, and motility during liquid storage. Poult. Sci..

[bib0013] Donoghue A.M., Wishart G.J. (2000). Storage of poultry semen. Anim. Reprod. Sci..

[bib0014] Ebeid T.A. (2012). Vitamin E and organic selenium enhances the antioxidative status and quality of chicken semen under high ambient temperature. Br. Poult. Sci..

[bib0015] Ebeid T.A. (2009). Organic selenium enhances the antioxidative status and quality of cockerel semen under high ambient temperature. Br. Poult. Sci..

[bib0016] El-Raghi A.A., Essawi W.M., Abdelnour S.A., El-Ratel I.T., Younis E.M., Abdel-Warith A.W.A., Gad A.M.A. (2024). Regulatory effects of vitamin E nano-emulsion on blood metabolites, immunological variables, testicular architecture, and sperm ultrastructure of heat-stressed V-line rabbit bucks. Ital. J. Anim. Sci..

[bib0017] Iaffaldano N., Rosato M.P., Manchisi A., Centoducati G., Meluzzi A. (2005). Comparison of different extenders on the quality characteristics of turkey semen during storage. Ital. J. Anim. Sci..

[bib0018] Iswati I., Isnaini N., Susilawati T. (2017). Fertilitas spermatozoa ayam buras dengan penambahan antioksidan glutathione dalam pengencer ringer's selama simpan dingin. J. Ilmu-Ilmu Peternak..

[bib0019] Jimoh O.A., Ayedun E.S., Ayodele S.O., Omoniyi S.I., Oladepo A.D., Lawal A.A., Ademola O.A., Kolawole B.J. (2021). Oxidative status and spermatozoa kinetics of rooster semen in citrus juice-based diluent. Trop. Anim. Health Prod..

[bib0020] Khalid A., Khudhair N., He H., Peng Z., Yaguang T., Guixue Z. (2016). Effects of dietary selenium supplementation on seminiferous tubules and SelW, GPx4, LHCGR, and ACE expression in chickentestis. Biol. Trace Elem. Res..

[bib0021] Koedkanmark T., Ratchamak R., Authaida S., Boonkum W., Semaming Y., Chankitisakul V. (2024). Supplementation of sperm cooling medium with Eurycoma longifolia extract enhances native Thai chicken sperm quality and fertility potential. Front. Vet. Sci..

[bib0022] Łukaszewicz E. (2000). Effects of semen filtration and dilution rate on morphology and fertility of frozen gander spermatozoa. Theriogenology.

[bib0023] Mann T. (1953). Inositol, a major constituent of the seminal vesicle secretion of the boar. Nature.

[bib0024] Marković R., Ciric J., Starčević M., Šefer D., Baltić M.Ž. (2018). Effects of selenium source and level in diet on glutathione peroxidase activity, tissue selenium distribution, and growth performance in poultry. Anim. Health Res. Rev..

[bib0025] Masoudi R., Sharafi M., Pourazadi L., Davachi N.D., Asadzadeh N., Esmaeilkhanian S., Dirandeh E. (2020). Supplementation of chilling storage medium with glutathione protects rooster sperm quality. Cryobiology.

[bib0026] Meamar M., Shahneh A.Z., Zamiri M.J., Zeinoaldini S., Kohram H., Hashemi M.R., Asghari S. (2016). Preservation effects of melatonin on the quality and fertility of native Fars rooster semen during liquid storage. Czech J. Anim. Sci..

[bib0027] Mohan J., Sharma S.K., Kolluri G., Dhama K. (2018). History of artificial insemination in poultry, its components and significance. World's Poult. Sci. J..

[bib0028] Nath A.K., Basu S., Datta U. (2015). Coenzyme Q10 and soyphosphatidylcholine in EK extender on preservation of Rhode Island Red poultry semen. J. Adv. Vet. Anim. Res..

[bib0029] Partyka A., Niżański W. (2021). Supplementation of avian semen extenders with antioxidants to improve semen quality—is it an effective strategy?. Antioxidants.

[bib0030] Pimpa J., Authaida S., Boonkum W., Rerkyusuke S., Janta C., Chankitisakul V. (2024). Unveiling the potential of *Aloe vera* gel supplementation in a cooling extender: a breakthrough in enhancing rooster sperm quality and fertility ability. Animals.

[bib0031] Pimprasert M., Kheawkanha T., Boonkum W., Chankitisakul V. (2023). Influence of semen collection frequency and seasonal variations on fresh and frozen semen quality in Thai native roosters. Animals.

[bib0032] Ratchamak R., Authaida S., Koedkanmark T., Boonkum W., Semaming Y., Chankitisakul V. (2024). Dietary supplementation with ginseng extract enhances testicular function, semen preservation, and fertility rate of mature and aging Thai native roosters. Theriogenology.

[bib0033] Ratchamak R., Authaida S., Koedkanmark T., Boonkum W., Semaming Y., Chankitisakul V. (2023). Supplementation of freezing medium with ginseng improves rooster sperm quality and fertility relative to free radicals and antioxidant enzymes. Animals.

[bib0034] Rehman Z.U., Meng C., Sun Y., Safdar A., Pasha R.H., Munir M., Ding C. (2018). Oxidative stress in poultry: lessons from the viral infections. Oxid. Med. Cell Longev..

[bib0035] Sahin K., Şahin E., Deeh P.B.D., Orhan V.K.C., Sahin N. (2023). Role of the antioxidant defence system and transcription factors in preventing heat stress in poultry: a dietary approach. Worlds Poult. Sci. J..

[bib0036] Silvestre M.A., Yániz J.L., Peña F.J., Santolaria P., Castelló-Ruiz M. (2021). Role of antioxidants in cooled liquid storage of mammal spermatozoa. Antioxidants.

[bib0037] Sonkusare S., Chaturvedi A. (2020). Glutathione: the master antioxidant and its role in health and disease. Am. J. Med. Sci. Pharm. Res..

[bib0039] Surai P.F., Wishart G.J. (1996). Strategies to improve poultry semen quality during preservation. Anim. Reprod. Sci..

[bib0040] Surai P.F., Fisinin V.I. (2014). Selenium in poultry breeder nutrition: an update. Anim. Feed. Sci. Technol..

[bib0041] Tvrdá E., Kňažická Z., Lukáčová J., Schneidgenová M., Goc Z., Gren A., Szabo C., Massányi P., Lukáč N. (2013). The impact of lead and cadmium on selected motility, prooxidant and antioxidant parameters of bovine seminal plasma and spermatozoa. J. Environ. Sci. Health A.

[bib0042] Uzochukwu I.E., Ali L.C., Amaefule B.C., Okeke C.C., Osita C.O., Machebe N.S., Yancheva V., Somogyi D., Nyeste K. (2025). Impact of vitamin E and selenium supplementation on growth, reproductive performance, and oxidative stress in dexamethasone-stressed Japanese quail cocks: vitamin E & selenium in stressed quail cocks. Poult. Sci..

[bib0043] Wróblewski M., Wróblewska W., Sobiesiak M. (2024). The role of selected elements in oxidative stress protection: key to healthy fertility and reproduction. Int. J. Mol. Sci..

[bib0044] Yaman M.A., Reza M.A., Abdullah A.N., Usman Y., Koesmara H. (2022). Sperm quality of hybrid chicken affected by Propolis, Honey, or Royal Jelly as organic diluent materials and storage periods during sperm preservation. Adv. Biol. Sci. Res..

[bib0046] Zanloo H., Soleimanzadeh A., Bucak M.N., Imani M., Zhandi M. (2022). The effects of glutathione supplementation on post-thawed Turkey semen quality and oxidative stress parameters and fertilization, and hatching potential. Theriogenology.

[bib0047] Zuha S., Rakha B.A., Akhter S., Ansari M.S., Waseem K. (2023). The effect of adding different levels of reduced glutathione to extender on the quality of cooled ring-necked pheasant semen. Biopreserv. Biobank..

