# Supplementary material for: Optimization of rooster semen preservation: a comparative study of extender types and antioxidant supplementation strategies during cold storage
Source: Poult Sci. 2025 Apr 4;104(6):105137. doi: 10.1016/j.psj.2025.105137 (PMC12005276; doi:10.1016/j.psj.2025.105137)
Supplement: Supplementary file 1 [file mmc1.pdf]

Certificate of Elsevier Language Editing Services

The following article was edited by Elsevier Language Editing Services:

Optimization of Rooster Semen Preservation: A Comparative Study of Extender Types and Antioxidant Supplementation Strategies During Cold Storage

Ordered by:

Vibuntita Chankitisakul

Estimated Delivery date:

2025-01-29

Order reference:

ASLESTD1093642
